# ADAM metallopeptidase domain 12 overexpression correlates with prognosis and immune cell infiltration in clear cell renal cell carcinoma

**DOI:** 10.1080/21655979.2021.2010313

**Published:** 2022-01-30

**Authors:** Junjie Gao, Dandan Yang, Haonan Xu, Kunpeng Yang, Jia Ma, Jun Xia, Xueshan Pan

**Affiliations:** a Bengbu Medical College Key Laboratory of Cancer Research and Clinical Laboratory Diagnosis, Bengbu Medical College, Anhui, Bengbu, China; b Department of Laboratory Medicine, Bengbu Medical College, Anhui, China; c Department of Clinical Medicine, Bengbu Medical College, Anhui, China; d Department of Biochemistry and Molecular Biology, School of Laboratory Medicine, Bengbu Medical College, Anhui, Bengbu, China

**Keywords:** ADAM12, clear cell renal carcinoma, prognosis, ceRNA, immune cells

## Abstract

This study investigated the role of ADAM metallopeptidase domain 12 (ADAM12) in clear cell renal cell carcinoma (ccRCC). The mRNA expression of ADAM12 was analyzed using The Cancer Genome Atlas (TCGA) database and the Gene Expression Omnibus (GEO) database, and the protein expression level of ADAM12 in renal clear cell carcinoma cell lines was detected by Western blot analysis. The Wilcoxon rank-sum test, logistic regression analysis, Cox regression analysis, and Kaplan-Meier analysis were used to assess the relationship between the clinicopathological characteristics and the prognosis of ccRCC patients and ADAM12 expression. The miRNAs and lncRNAs associated with ADAM12 were predicted, and a ceRNA network was constructed using the Starbase database. Gene Set Enrichment Analysis (GSEA) and Gene Ontology (GO) analysis were used to identify relevant pathways. The relationship between ADAM12 and immune infiltration and checkpoints was analyzed using the TIMER and Gene Expression Profiling Interactive Analysis (GEPIA) databases. The results showed that ADAM12 expression was increased in ccRCC tissues and cells and significantly correlated with patient gender, Tumor stage, Metastasis stage, Node stage, and clinical grade. Survival analysis showed that ccRCC patients with high ADAM12 expression had a low overall survival rate. Univariate and multivariate Cox regression analyses showed that ADAM12 was an independent prognostic factor. Enrichment analysis showed that ADAM12 expression was associated with immune-related pathways. Immune infiltration analysis showed that ADAM12 expression was related to immune cell infiltration, PD-1, PD-L1, and CTLA4. These results suggest that ADAM12 may be a potential diagnostic and prognostic biomarker for ccRCC.

## Introduction

According to the results of recent studies, the incidence and mortality of patients with renal cancer are both increasing [1,2]. Clear cell renal cell carcinoma (ccRCC) accounts for 75–80% of primary renal cancer [3], which is mainly characterized by chromosome 3p deletion, resulting in uncontrolled hypoxia-inducible factor (HIF) due to von Hippel Lindau (VHL) gene dysfunction [4]. Surgical treatment remains the essential treatment for ccRCC due to the poor sensitivity of renal clear cell carcinoma to chemotherapy and radiotherapy [5]. However, the tendency of ccRCC for recurrence and metastasis maintains the mortality of patients at persistently high rates [6]. Therefore, finding potential biomarkers for the early diagnosis of renal clear cell carcinoma has become significant.

ADAM12, also known as disintegrin and metalloproteinase domain-containing protein 12, is located on chromosome 10q26.2, and two splice variants, ADAM12-L and ADAM12-S, the transmembrane and secreted forms of ADAM12, have been reported [7]. Studies have shown that ADAM12 expression was elevated in breast cancer [8,9], cervical cancer [10], ovarian cancer [11], lung cancer [12], prostate cancer [13], colorectal cancer [14,15], and glioma [16], and elevations in ADAM12 were correlated with malignant biological behavior such as the proliferation, migration, and invasion of tumor cells. However, there are few reports on ADAM12 in ccRCC, so this study aimed to explore the biological function and prognostic value of ADAM12 in ccRCC.

Non-coding RNAs (ncRNAs) have been studied in a large number of diseases [17], among which lncRNAs have gained much attention as a type of non-coding RNA, which can participate in tumor proliferation, metastasis, migration, and apoptosis characteristics, indicating that lncRNAs can be potential targets for cancer therapy [18]. The competing endogenous RNA (ceRNA) hypothesis suggests [19] that lncRNA can act as an endogenous ceRNA to bind miRNA and promote mRNA expression competitively. This competitive binding effect is also known as the miRNA sponge effect [20,21]. The ceRNA network is closely related to the occurrence and development of liver cancer [22], breast cancer [23], and pancreatic cancer [24]. It has been shown that the SNHG4/miR-204-5p/RUNX2 axis could influence the progression of ccRCC [25]. And the increased expression of LncRNA ARSR was shown to affect the overall survival of ccRCC patients [26]. LncRNA 00312 was shown to inhibit the expression of miR-34a-5p *in vitro*, suppressing the proliferation and migration of ccRCC cells [27]. The above studies suggest that the ceRNA network may be involved in ccRCC development and progressing [28]. However, the ceRNA network significantly associated with ccRCC prognosis still needs further investigation.

In recent years, tumor immunotherapy approaches such as immune checkpoint blockade (ICB) and chimeric antigen receptor T cell (CAR-T) therapy have made great strides and completely changed the treatment modalities for tumors [29]. Immunotherapy is being used in the clinic for the treatment of ccRCC [30]. Although immune checkpoint blockade technology has achieved durable disease control in some patients with advanced ccRCC, the molecular mechanisms underlying ICB therapy for ccRCC are not fully understood [31]. Therefore, it is necessary to explore and investigate the relationship between the immune checkpoint blockade response and the ceRNA network in ccRCC.

**Figure 1.**
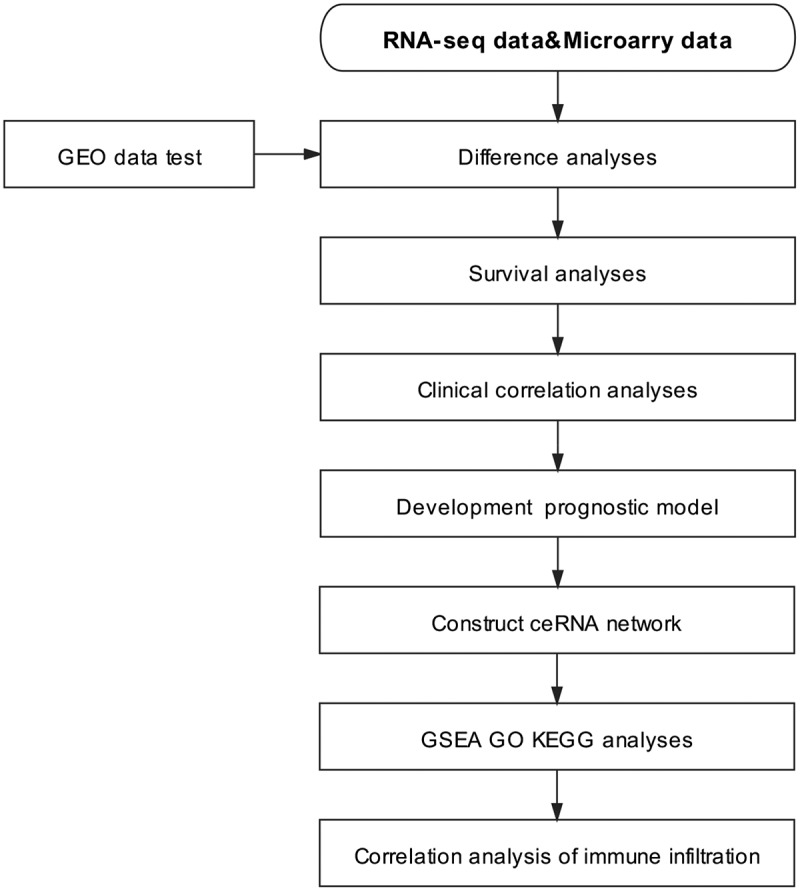
Experimental technical roadmap.

As stated by Kumar, bioinformatics integration and the analysis of massive sequencing data helps to identify gene regulatory pathways as well as networks in diseases [32], allows for the comparison of differentially expressed genes in patients with different diseases [33], and has become a common technique to study the pathogenesis of complex diseases [34].In this study, ccRCC data were obtained from The Cancer Genome Atlas (TCGA), which contains RNA expression profiling data from 539 tumor samples and 71 nontumor samples. The Gene Expression Omnibus (GEO) datasets GSE53757, GSE46699, and GSE66271 were used as validation sets. To validate the potential biological function and prognostic value of ADAM12 in ccRCC by bioinformatics means, we simultaneously screened the miRNAs and lncRNAs upstream of ADAM12, constructed a ceRNA network, and investigated whether the network might play a regulatory role in the development of ccRCC. Finally, we performed enrichment and correlation analyses. The flow chart of the study is shown in Figure 1. Through the above analysis, we found that ADAM12 expression was elevated and significantly different in ccRCC. Hence, we speculated that ADAM12 expression might be associated with the progression and prognosis of patients with ccRCC. This study aimed to investigate the prognostic value of ADAM12 in clear cell carcinoma of the kidney and comprehensively evaluate its potential value. The results were preliminarily verified by Western blot analysis.

## Materials and methods

### Cell sources and cell culture

The human renal clear cell carcinoma cell lines 786-O, caki-2, 769-P, and human normal renal epithelial 293 T cells were purchased from the cell bank of the Chinese Academy of Sciences (Shanghai, China). 786-O cells and 769-P cells were cultured in RPMI-1640 medium (Gibco, Gaithersburg, MD, USA) supplemented with 10% fetal bovine serum (FBS) and placed at 37°C and 5% carbon dioxide. 293 T cells were cultured in Dulbecco’s modified Eagle’s medium (DMEM) (Gibco) supplemented with 10% FBS and placed at 37°C and 5% carbon dioxide. Caki-2 cells were cultured in McCoy’s 5A modified medium (Gibco) supplemented with 10% FBS and placed at 37°C and 5% CO_2_.

### Sample information collection

Expression profiling data and clinical information on the human clear cell renal cell carcinoma samples were downloaded from TCGA database (https://portal.gdc.cancer.gov/. The expression data of mRNA and lncRNA (tumor = 539, normal = 72) and miRNA (tumor = 545, normal = 71) were included. All the raw RNA sequence data (lncRNAs, miRNAs, and mRNAs) were obtained. Normalization was performed to obtain the expression profile input files. Gene expression profiles were downloaded from the National Center for Biotechnology Information Public Gene Chip data platform (GEO) database (https://www.ncbi.nlm.nih.Gov/GEO/): GSE53757 (including 72 ccRCC samples and 72 adjacent nontumor samples), GSE46699 (including 67 ccRCC samples and 63 adjacent nontumor samples), and GSE66271 (including 13 ccRCC samples and 13 adjacent nontumor samples). The UCSC (https://xenabrowser.net/datapages/) database was used to download expression data on additional tumors including urothelial bladder carcinoma, breast cancer, cholangiocarcinoma, colorectal cancer, esophageal cancer, glioma, head and neck squamous cell carcinoma, renal chromophobe carcinoma, renal papillary cell carcinoma, liver cancer, lung adenocarcinoma, lung squamous cell carcinoma, prostate cancer, rectal adenocarcinoma, gastric cancer, thyroid cancer, and endometrial cancer, and survival data were retrieved from the Starbase (http://starbase.sysu.edu.cn/) database [35] to obtain the related miRNA and lncRNA results. Immune infiltration-related outcomes were obtained from the TIMER database [36–38] (http://timer.comp-genomics.org/) and immune checkpoint correlation analysis results were obtained from the GEPIA database [39] (http://gepia.cancer-pku.cn/).

### Expression difference analysis

The RNAseq data obtained from TCGA and the GEO downloads were name-transformed and log2 corrected to generate the expression of ADAM12 in the tumor group and the adjacent nontumor group. The expression of ADAM12 in the two groups was statistically analyzed using the R software ggplot2 package [40], limma package [41], and beeswarm package [42], and scatter and pairwise plots were generated to visualize the results. A *p*-value of < 0.05 was considered statistically significant.

### Survival analysis

The patients were divided into high-expression ADAM12 and low-expression ADAM12 groups according to the median ADAM12 gene expression in ccRCC patients. The statistical analysis of patient survival data was performed using the R software survival package [43], and the survminer package [44] was used to visualize the analysis results. A *p-*value of < 0.05 was considered to indicate significance for patient survival prognosis.

### Analysis of clinical relevance

The clinical data of the ccRCC patients were downloaded from TCGA database, and the patient clinical information and ADAM12 expression data were combined. Then, the R software ggpubr package [45] was used for statistical analysis and the visualization of patient age, sex, clinical grade, T stage, M stage, and N stage.

### Univariate multivariate regression analysis

To investigate the role of ADAM12 expression in ccRCC patients, we used univariate Cox regression analysis to calculate the correlation between ADAM12 expression levels and patient survival. Multivariate analysis was then employed to evaluate whether ADAM12 was an independent prognostic factor for ccRCC patient survival. A *p-*value o*f* < 0.05 in Cox regression analysis indicated statistically significant ADAM12 expression.

### Construction of the ceRNA network

The Starbase database was utilized. The mRNA upstream miRNAs were selected, and the resulting miRNAs were subjected to correlation analysis, expression difference analysis, and survival analysis and visualized using the ggpubr package [45], ggExtra package [46], and survival package [43] in R software and the resulting miRNAs were selected to meet the criteria of low ADAM12 mRNA expression in tumor tissues, positive correlation with the prognosis of patients, and negative correlation with expression with statistical significance in the patients. Increases upstream of the miRNAs were screened using the Starbase database, and the obtained lncRNAs were selected to meet the requirements of negative correlation with miRNA expression and positive correlation with mRNA expression, and higher expression in tumor tissues and negative correlation with patient survival time. The above differences were statistically significant. The resulting lncRNA, miRNA, and mRNA networks were imported into Cytoscape 3.8 to draw a Cerna network diagram.

### GSEA (Gene Set Enrichment Analysis) enrichment analysis

GSEA is a commonly used bioinformatics analysis method to determine whether a previously defined set of genes shows statistically significant, consistent differences between two phenotypes [47]. The mRNA expression data of 539 tumor tissues obtained from TCGA database were divided into high and low groups according to the median ADAM12 expression. Then, the grouping file and expression matrix file were imported into gsea4.1 software for enrichment analysis and exported to elucidate the significant functional and pathway differences between the high and low ADAM12 groups. One thousand gene set permutations were performed for each analysis. After correction, a *p*-value of < 0.05, false discovery rate (FDR) of < 0.25, and normalized enrichment scores (|NES|) of > 1 were considered significantly enriched.

### GO (Gene Ontology) and KEGG (Kyoto Encyclopedia of Genes and Genomes) enrichment analysis

To understand the possible biological processes and pathways involved in ADAM12, we used logFC > |1|, *p* < 0.05 as the screening condition to obtain the genes related to ADAM12 expression, then, the R software clusterProfiler package [48], org.hs.eg.db package, enrichplot package, ggplot2 package, and the GO plot package were used for GO and KEGG enrichment analysis and visualization.

### Immune correlatative analysis

To investigate the relationship between ADAM12 expression and tumor-infiltrating immune cells, we used the R software ggpubr package [45] and ggextra package [46] to analyze the correlation between ADAM12 expression and immune cell (including B cells, CD4^+^T cells, CD8^+^T cells, neutrophils, macrophages, and dendritic cells) checkpoints using TIMER. Online website analysis of ADAM12 and immune cell infiltration was visualized while applying GEPIA (http://gepia.cancer-pku.cn/) Online website to analyze the correlation between ADAM12 and immune checkpoints.

### Western blotting analysis

Cells were lysed with RIPA lysis buffer (Beyotime, China) mixed with protease inhibitors (Beyotime) under ice bath conditions for 30 min. Protein concentration was determined using the BCA Protein Quantification Kit (Beyotime). Proteins (50 μg) were separated by 10% sodium dodecyl sulfate-polyacrylamide gel electrophoresis and transferred onto polyvinylidene fluoride (PVDF) membranes (Millipore, Bedford, MA, USA). The membranes were blocked in 5% nonfat milk for 2 h at room temperature and incubated with primary antibodies overnight at 4֩C. The membranes were incubated with horseradish peroxidase-labeled secondary antibodies (1:2000; Lianke bio, Hangzhou, China) for 2 h at room temperature, and β-actin was used as a protein loading control. Finally, an electrochemiluminescence detection system was used for visualization. The source of the antibodies and the concentrations used were rabbit anti-ADAM12 (1:1000, Abclonal, China) and β-actin (1:2000, Abclonal).

### Statistical analyses

Statistical analyses were performed using SPSS (version 26.0) and R (version 4.1.0, https://www.R-project.org/). The Wilcoxon signed-rank test and Wilcoxon rank-sum test were performed to investigate the expression of ADAM12 in paired and unpaired samples, respectively. The Wilcoxon signed-rank test was used to analyze the relationship between ADAM12 expression and clinical characteristics. Univariate and multivariate analyses were performed using Cox regression models to assess the risk of death, including gender, age, T stage, N stage, M stage, pathological stage, and ADAM12 expression. A *p-*value of < 0.05 was considered statistically significant.

## Results

This study concluded that ADAM12 expression was significantly elevated in ccRCC tissues using data from TCGA and GEO databases in a comprehensive analysis. Follow-up data showed that overall survival was significantly lower in patients with high ADAM12 expression and positively correlated with clinical stage and grade. Univariate and multifactorial regression analyses suggested that ADAM12 may be a potential prognostic marker. Pathway enrichment analysis indicated that ADAM12 was involved in the development of ccRCC through immune-related pathways, and further investigation revealed that ADAM12 expression was associated with immune cell infiltration into tumors. The above results suggest that ADAM12 can be used as a diagnostic and prognostic indicator for renal clear cell carcinoma.

### Patient characteristics

Our data were obtained from TCGA, including 539 ccRCC patients with available clinical data and gene expression data. Clinical characteristics including gender, age, histological grade, pathological grade, T stage, N stage, M stage, treatment outcome, and vital status were collected (Table 1).

**Table 1.** Clinical characteristics of ccRCC patients in TCGA

Characteristic	levels	Overall
n		539
Gender, n (%)	Female	186 (34.5%)
	Male	353 (65.5%)
Age, n (%)	≤60	269 (49.9%)
	>60	270 (50.1%)
Histologic grade, n (%)	G1	14 (2.6%)
	G2	235 (44.3%)
	G3	207 (39%)
	G4	75 (14.1%)
Pathologic stage, n (%)	Stage I	272 (50.7%)
	Stage II	59 (11%)
	Stage III	123 (22.9%)
	Stage IV	82 (15.3%)
Tumor stage, n (%)	T1	278 (51.6%)
	T2	71 (13.2%)
	T3	179 (33.2%)
	T4	11 (2%)
Node stage, n (%)	N0	241 (93.8%)
	N1	16 (6.2%)
Metastasis stage, n (%)	M0	428 (84.6%)
	M1	78 (15.4%)
Primary therapy outcome, n (%)	PD	11 (7.5%)
	SD	6 (4.1%)
	PR	2 (1.4%)
	CR	128 (87.1%)
OS event, n (%)	Alive	366 (67.9%)
	Dead	173 (32.1%)

### ADAM12 expression is elevated in renal clear cell carcinoma and associated with poor prognosis

**Figure 2.**
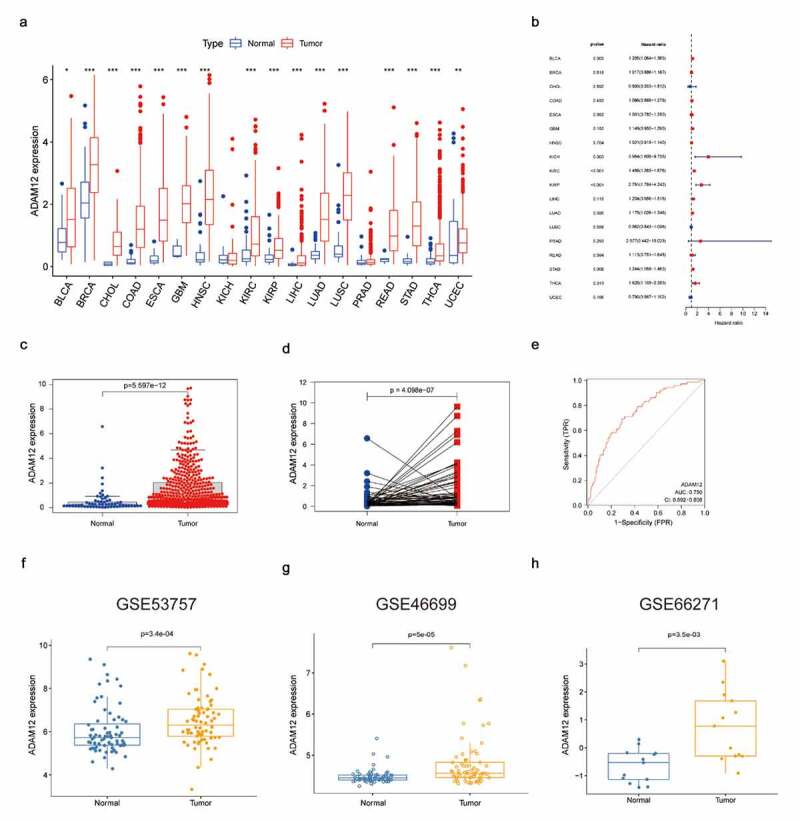
Significantly elevated expression levels of ADAM12 in renal clear cell carcinoma samples.

The expression of ADAM12 in different types of tumors was analyzed based on TCGA database and ADAM12 expression was found to be increased in the vast majority of tumors, with statistically significant differences (Figure 2a). Analysis of the clinical prognostic data of patients with different tumors revealed a good correlation between the increased expression of ADAM12 in RCC and poor patient prognosis (Figure 2b). The diagnostic value of ADAM12 mRNA expression was evaluated by receiver operating characteristic (ROC) curve analysis in TCGA datasets. ADAM12 expression in tumor tissue was significantly higher than that in the normal tissues (*p* = 5.597e-12) (Figure 2c) and pairing the results between cancer and adjacent normal tissues also confirmed that ADAM12 expression in the tumor group was higher than that in the adjacent control group (*p* = 4.098e-7) (Figure 2d) ADAM12 had some diagnostic value for renal clear cell carcinoma (Figure 2e). Consistently, analysis in the three GEO datasets yielded a higher expression of ADAM12 in the tumor group (figure 2f-h).

(A) ADAM12 expression is elevated in different tumor tissues. (B) ADAM12 expression and overall survival of patients with different tumors (C) ADAM12 expression was elevated in ccRCC based on TCGA dataset. (D) ADAM12 expression was elevated in ccRCC and the paired adjacent tissues based on TCGA dataset. (E) ADAM12 expression elevations had diagnostic value based on ccRCC receiver operating characteristic analysis (AUC = 0.75). (F-H) ADAM12 expression was elevated in ccRCC based on the GEO dataset GSE53757, GSE46699, and GSE66271 (*p < 0. 05, **p < 0.01, ***p < 0.001).

### ADAM12 correlates with clinical features in patients with clear cell renal cell carcinoma

**Figure 3.**
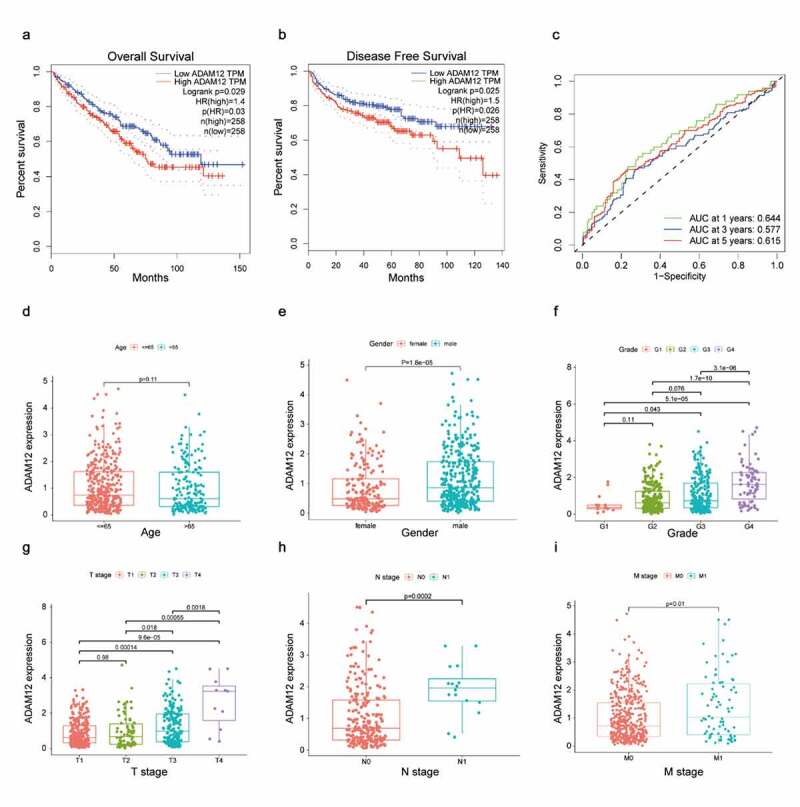
ADAM12 expression correlates with the prognosis and clinicopathologic characteristics of ccRCC patients.

The Kaplan-Meier survival curve showed that patients with high ADAM12 expression had shorter survival times than those with low ADAM12 expression (*p* = 0.029), and patients with high ADAM12 expression also had shorter disease-free survival time than those with low ADAM12 expression (*p* = 0.025) (Figure 3a, b)The diagnostic value of ADAM12 mRNA expression on 1 -, 3 -, and 5-year patient survival rates were evaluated by ROC curves, and the area under the curve (AUC) was 0.644, 0.577, and 0.764, respectively (Figure 3c). The expression of ADAM12 mRNA in each group was observed according to the patients’ age, sex, clinical grade, T stage, N stage, and M stage. ADAM12 expression was independent of age (Figure 3d). However, it was significantly higher in male patients (*p* = 1.8e-5) than in female patients (*p* = 1.8e-5) (Figure 3e). Patients with a high clinical grade (G3/G4) had higher ADAM12 mRNA expression levels than those with low clinical grade (G1/G2) (figure 3f). The same results were obtained for the T stage, M stage, and N stage (Figure 3g-i).

(A) The overall survival time of ccRCC patients with high ADAM12 expression was significantly shorter than that of ccRCC patients with low ADAM12 expression based on TCGA dataset. (B) The disease-free survival time of ccRCC patients with high ADAM12 expression was significantly shorter than that of ccRCC patients with low ADAM12 expression based on TCGA dataset. (C) Receiver operating characteristic curves for survival showed that ADAM12 had a good predictive value for 1-year survival (AUC = 0.644). (D-I) ADAM12 expression was positively correlated with sex, clinical grade, T stage, N stage, and M stage but not with age.

### Development of a prognostic model based on ADAM12 and clinical factors

**Figure 4.**
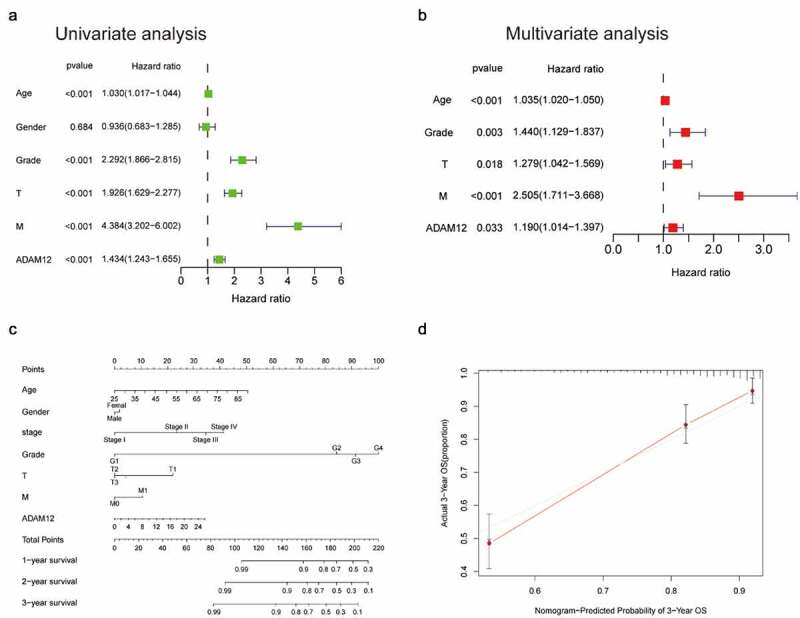
Nomogram and calibration curve for predicting the overall probability of survival in ccRCC patients.

Univariate regression analysis revealed that ADAM12 mRNA expression was correlated with tumor grade, T stage, and M stage, as well as overall survival (Figure 4a). Multivariate analysis revealed that ADAM12 mRNA expression was an independent risk factor for overall survival in patients with clear cell renal cell carcinoma (Figure 4b). We constructed a predictive model for overall survival by fitting the expression of ADAM12 and other clinical parameters, establishing a nomogram to integrate ADAM12 as a ccRCC biomarker (Figure 4c) with higher points on the nomogram representing worse prognostic factors. A calibration curve evaluated the performance of the nomogram for ADAM12 expression versus 3-year survival (Figure 4d).

(A) Univariate analysis showed that grade, T-stage, M-stage, and ADAM12 expression levels were independent predictors of survival time in ccRCC patients. (B) Multivariate analysis showed that ADAM12 expression was a reliable prognostic predictor for ccRCC patients. (C) Nomogram integrating ADAM12 and other ccRCC prognostic factors based on TCGA data. (D) Calibration curve of the nomogram (C-index = 0.7221).

### Construction of lncRNA-miRNA-mRNA triple regulatory network

**Figure 5.**
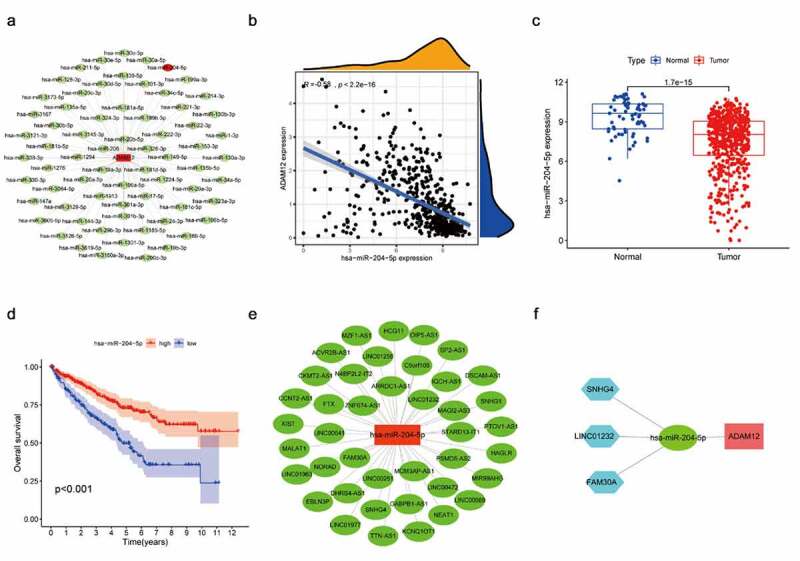
Screening of ADAM12 upstream miRNAs.

**Figure 6.**
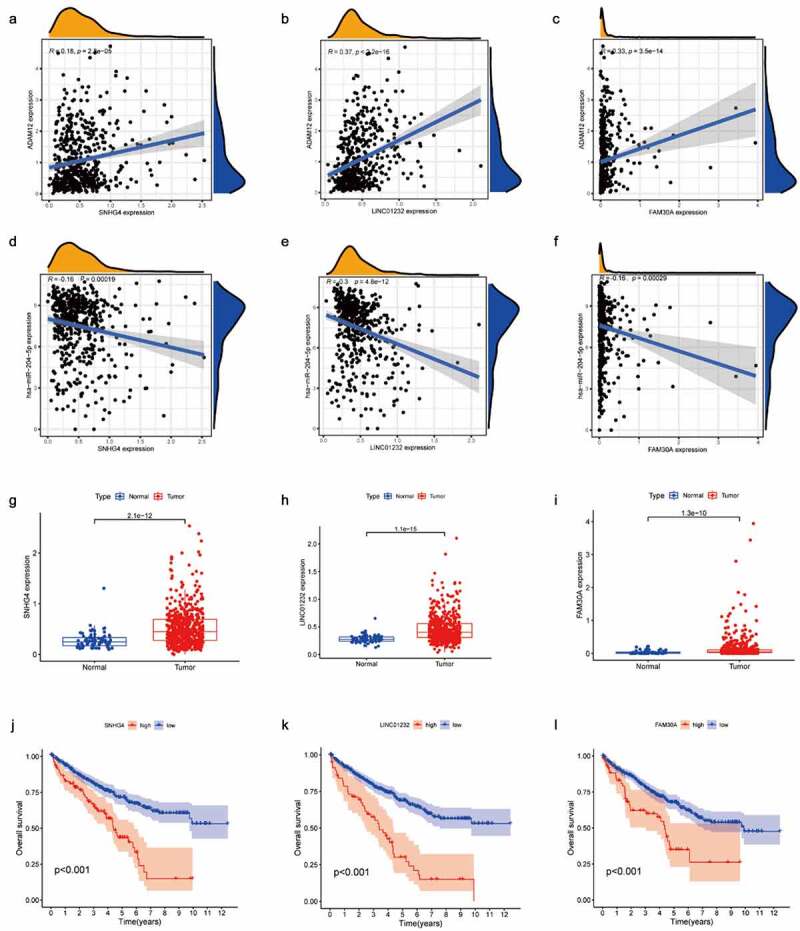
Screening of has-miR-204-5p upstream lncRNAs.

Through the ceRNA hypothesis, we know that miRNA can bind to mRNA as a negative regulator to regulate mRNA expression, and lncRNA as a competitive endogenous RNA can bind to miRNA to indirectly regulate mRNA expression. Based on the above theory, we hoped to screen the miRNA and lncRNA upstream of ADAM12 using bioinformatics means and thus, construct a ceRNA network. Through the Starbase number, library screening yielded 69 miRNAs associated with ADAM12 (Figure 5a). We screened out one miRNA, namely hsa-mir-204-5p, which was negatively correlated with ADAM12 expression and lowly expressed in renal clear cell carcinoma. The expression level of hsa-mir-204-5p was positively correlated with the survival time of patients (Figure 5b-d). lncRNA upstream of the miRNA was screened by the same method (Figure 5e). Moreover, the upstream lncRNA meeting the criteria of negative correlation with has-mir-204-5p expression was positively correlated with ADAM12 expression, which was elevated in renal clear cell carcinoma, and the expression was negatively correlated with patient survival time (Figure 6). Finally, three lncRNAs, SNHG4, LINC01232, and FAM30A, were obtained, and a ceRNA regulatory network was constructed (figure 5f).

(A) Network mapping of ADAM12-associated miRNAs based on the Starbase database. (B) ADAM12 expression was negatively correlated with has-miR-204-5p expression based on TCGA dataset. (C) Has-miR-204-5p expression was reduced in ccRCC tissues based on TCGA dataset. (D) The overall survival of ccRCC patients with high expression of has-miR-204-5p was associated with longer overall survival than ccRCC patients with low expression of has-miR-204-5p. (E) Mapping of has-miR-204-5p-related lncRNA network based on the Starbase database. (F) Construction of the ADAM12-centered ceRNA network.

(A-C) ADAM12 expression was positively correlated with SNHG4, LINC01232, and FAM30A expression. (D-E) Has-miR-204-5p expression was negatively correlated with SNHG4, LINC01232, and FAM30A expression. (G-I) SNHG4, LINC01232, and FAM30A expression was elevated in ccRCC based on TCGA dataset. (J-L) Overall survival time was shorter in ccRCC patients with high expression of SNHG4, LINC01232, and FAM30A than in ccRCC patients with low expression of SNHG4, LINC01232, and FAM30A based on TCGA dataset.

### ADAM12 GSEA enrichment score, GO, and KEGG enrichment analysis

**Figure 7.**
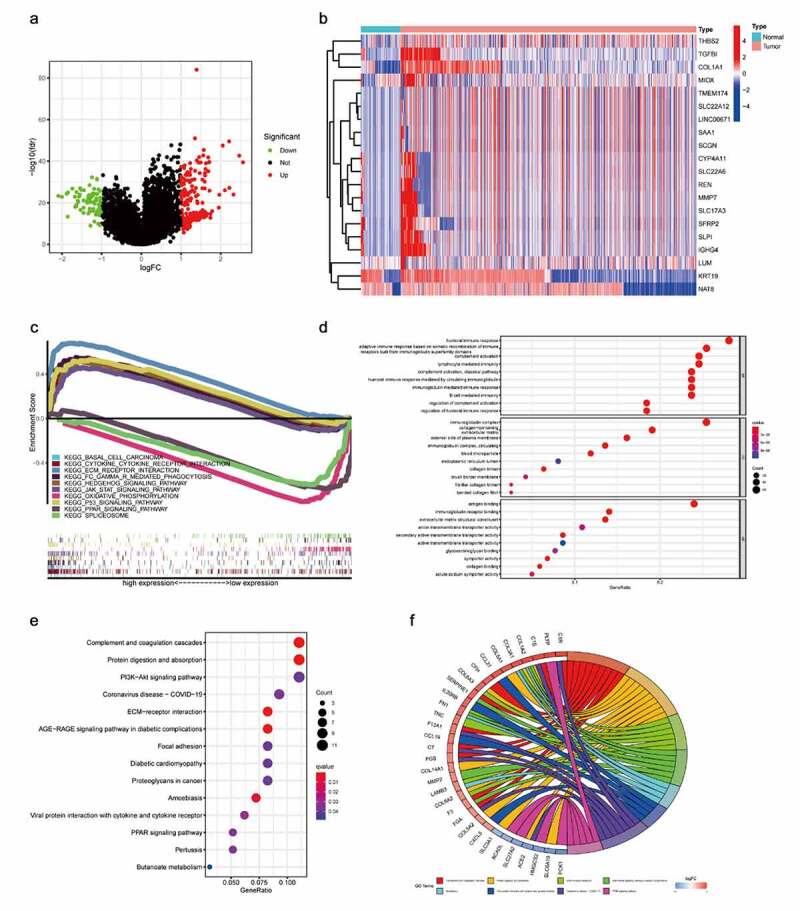
Gene enrichment analysis to identify ADAM12-associated pathways.

Screening genes highly correlated with ADAM12 in renal clear cell carcinoma resulted in 250 associated genes, of which 178 were upregulated and 72 were downregulated (Figure 7a). Twenty significantly differentially expressed genes were selected for analysis, and the upregulated genes were highly expressed in tumors, which was consistent with the ADAM12 expression results. In contrast, the downregulated genes showed decreased expression in tumors (Figure 7b). GSEA enrichment analysis was used to identify the signaling pathways associated with ADAM12 mRNA expression in ccRCC. The results showed that cytokine-cytokine receptor interaction, ECM receptor interaction, and FC-γ-R-mediated phagocytosis were correlated with ADAM12 expression (Figure 7c). GO and KEGG enrichment analyses were performed for the related genes. ADAM12-related genes were mainly associated with the humoral immune response and the adaptive immune response based on the somatic recombination of immune receptors constructed by the immunoglobulin superfamily domain, immunoglobulin complexes, and ADAM12-related genes were mainly associated with complement and coagulation cascade, protein digestion and uptake, and PI3K-Akt signaling pathway by KEGG enrichment analysis (Figure 7d-f).

(A) Screening of genes positively or negatively associated with ADAM12 expression to plot volcanoes, with red dots indicating genes positively associated with ADAM12 expression and green dots indicating genes negatively associated with ADAM12 expression. (B) Heatmap of genes associated with ADAM12 expression showing only the top 20. (C) Gene Set Enrichment Analysis (GSEA) showed that cytokine-cytokine receptor interaction, ECM receptor interaction, and FC-γ-R-mediated phaγocytosis were correlated with ADAM12 expression. (D) Gene Ontology (GO) enrichment analysis indicated that ADAM12 was associated with the humoral immune response. (E, F) Kyoto Encyclopedia of Genes and Genomes (KEGG) enrichment analysis indicated that ADAM12 was associated with the PI3K Akt signaling pathway.

### Correlation between ADAM12 expression and immune infiltration in clear cell carcinoma of the kidney

**Figure 8.**
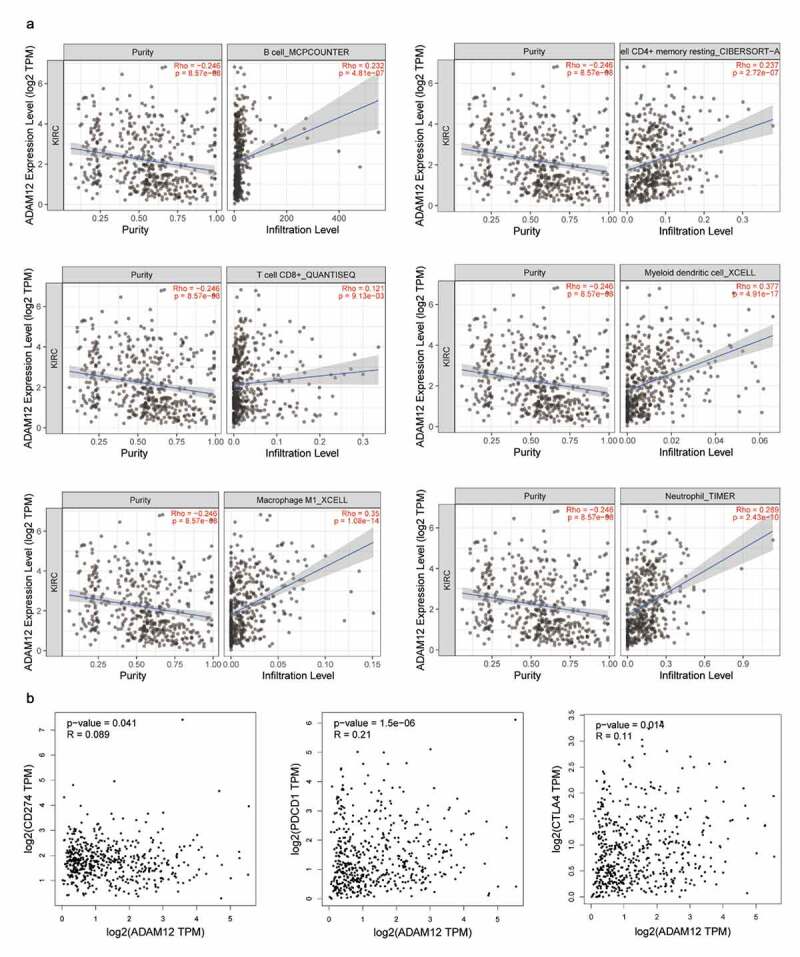
Correlation between the infiltration of immune cells, PDCD1 (PD1), CD274 (PD-L1), and CTLA4 and ADAM12 expression in ccRCC.

Through GSEA enrichment analysis and GO and KEGG enrichment analyses, ADAM12 was mainly associated with the immune response. Analysis of the correlation between ADAM12 and the gene signature of immune cells (Table 2) revealed a significant positive correlation between ADAM12 and the expression of six immune response markers on B cells, CD4^+^T cells, CD8^+^T cells, M2-type macrophages, and neutrophils, as well as dendritic cells. The correlation analysis between ADAM12 and immune cell infiltration yielded consistent results (Figure 8a). A positive correlation between ADAM12 expression and the immune checkpoints PD1, PDL-1, and CTLA4 was obtained by correlation analysis between ADAM12 and immune checkpoint genes (CD274, PDCD1, and CTLA4) (Figure 8b).

**Table 2.** The association between the expression level of ADAM12 and immune cell infiltration in the tumor microenvironment

Immune Cell	Gene	Correlation	*p* value
B cell	CD19	0.37679961	0
B cell	CD79A	0.329669332	6.68E-15
CD8^+^ T cell	CD8A	0.132687633	0.002117302
CD8^+^ T cell	CD8B	0.122331324	0.004626455
CD4^+^ T cell	CD4	0.375355502	0
M1 macrophage	NOS2	0.028626609	0.508668845
M1 macrophage	IRF5	−0.023321855	0.590293363
M1 macrophage	PTGS2	0.361055324	1.95E-18
M2 macrophage	CD163	0.432917869	0
M2 macrophage	VSIG4	0.483661395	0
M2 macrophage	MS4A4A	0.412962568	0
Neutrophil	CEACAM8	−0.002170124	0.96006042
Neutrophil	ITGAM	0.302640191	1.11E-12
Neutrophil	CCR7	0.333767031	2.88E-15
Dendritic cell	HLA-DPB1	0.116755114	0.006888812
Dendritic cell	HLA-DQB1	−0.022688746	0.600423222
Dendritic cell	HLA-DRA	0.119807113	0.005550931
Dendritic cell	HLA-DPA1	0.107351644	0.013008619
Dendritic cell	CD1C	0.110131444	0.01079865
Dendritic cell	NRP1	0.177258436	3.84E-05
Dendritic cell	ITGAX	0.2045146	1.94E-06

(A) ADAM12 expression was significantly and positively correlated with the infiltration of B-cells CD4^+^T cells, CD8^+^T cells, dendritic cell macrophages, and neutrophils. (B) ADAM12 expression was positively correlated with CD274, PDCD1, and CTLA4 expression.

### Expression profile of ADAM12 in normal renal epithelial cells as well as renal cancer cells

**Figure 9.**
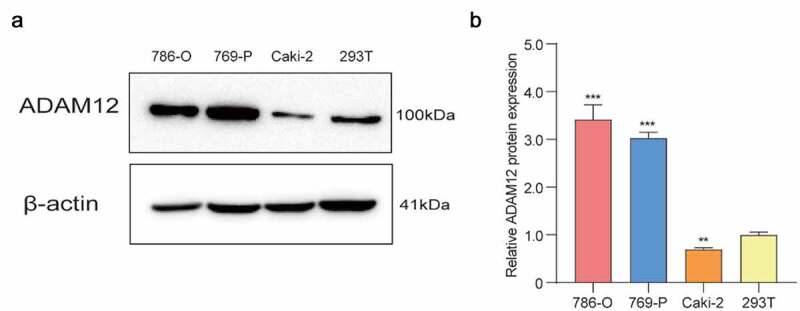
Elevated expression of ADAM12 in renal clear cell carcinoma cell lines.

The bioinformatics-based analysis found that ADAM12 expression was elevated in ccRCC tissues and an *in vitro* cell-based assay to validate the expression of ADAM12 protein in ccRCC cell lines and renal epithelial cells. We found that the protein expression of ADAM12 was low in 293 T but high in 786-O and 769-P renal clear cell carcinoma cell lines (Figure 9). This was consistent with the results of ADAM12 mRNA expression in tissues derived from bioinformatics analysis of RNAseq.

(A) Western blot assay of ADAM12 protein expression in three renal clear cell carcinoma cell lines 786-O, 769-P, and Caki-2, and one normal renal epithelial cell line 293 T, showing the elevated expression of ADAM12 in renal clear cell carcinoma cell lines. (B) Relative quantitative results of Western blots (**p < 0.01, ***p < 0.001).

### Discussion

ccRCC is a common urologic tumor [1] Since renal clear cell carcinoma is not sensitive to current chemotherapy and radiotherapy [49],molecular targeted therapy and immunotherapy are emerging as essential treatments for ccRCC. However, due to the lack of specific molecular targets, the efficacy of molecular targeted therapy and immunotherapy in patients with advanced ccRCC remains unsatisfactory [50] Therefore, it becomes essential to find biomarkers for early diagnosis and molecular targets for immunotherapy. We searched for genes associated with the development and prognosis of renal clear cell carcinoma using bioinformatics. We searched for upstream lncRNAs and miRNAs based on key mRNAs. Finally, we obtained a ceRNA network associated with ccRCC immune infiltration and prognosis to explore ccRCC prognostic biomarkers further, and potential therapeutic targets provide clues.

The results of our analysis showed that ADAM12 expression was elevated in renal clear cell carcinoma, and ccRCC patients with high ADAM12 expression had significantly shorter survival than those with low ADAM12 expression. Further analysis of the clinical data of ccRCC patients showed that ADAM12 expression was correlated with the tumor grade, T stage, M stage, and N stage of ccRCC patients, and high ADAM12 expression was found to be an independent factor associated with shorter survival in univariate and multivariate analyses. The above analyses indicated that ADAM12 might be a poor prognostic gene in ccRCCs, and patients with high ADAM12 expression tended to have shorter survival. The tumor grade and T, M, and N stages tended to be more posterior, which was also consistent with the results of ADAM12 in other tumors studied by other authors, indicating that ADAM12 can be a potential biomarker for ccRCC.

Based on the ceRNA hypothesis, we continued to explore the miRNAs and lncRNAs upstream of ADAM12. We screened out miRNAs upstream of ADAM12, namely hsa-mir-204-5p, by combining correlation analysis with the Starbase database. Numerous studies have shown that mir-204-5p may function as a tumor suppressor by inhibiting the proliferation, migration, and invasion of tumor cells [51–57]. However, our study also found that ccRCC patients with a decreased expression of miR-204-5P and low expression of miR-204-5P had significantly shorter survival times than those with a high expression of miR-204-5P, which was also negatively correlated with ADAM12 expression with a high correlation coefficient, indicating that miR-204-5P may target ADAM12 in ccRCC mRNA, thereby inducing its degradation. Based on the miRNA sponge theory, we obtained the miRNA upstream lncRNAs, namely SNHG4, LINC01232, and FAM30A, and these three lncRNAs were highly expressed in ccRCC and associated with poor prognosis. An lncRNA-miRNA-mRNA ceRNA network was constructed based on the expression of ADAM12 mRNA. The analysis of this network preliminarily suggested the pathway in which ADAM12 played a role in ccRCC.

GSEA enrichment analysis indicated that ADAM12 might be associated with the cytokine-cytokine receptor interaction pathway, the ECM receptor interaction pathway, FC-γ-R-mediated phagocytosis, cytokines, ECM receptors, and the FC-γ-R, which are common immune regulatory pathways Previous studies also indicated that ADAM12 might play a role in lung adenocarcinoma, and pancreatic cancer through immune pathways. Previous studies also showed that ADAM12 may play a role in lung adenocarcinoma, pancreatic cancer, and other tumors through immune pathways [58,59]. To determine whether ADAM12 could also regulate ccRCC through immune-related pathways, GO functional enrichment analysis and KEGG pathway enrichment analysis of ADAM12-associated genes were performed. The results indicated that the primary biological processes involved in ADAM12 were the humoral immune response and the adaptive immune response based on the somatic recombination of immune receptors constructed by the immunoglobulin superfamily domain and complement activation. These biological processes were all related to the immune response. KEGG enrichment analysis further illustrated the association of ADAM12 with complement and coagulation cascades, ECM receptor interaction, and the PI3K Akt signaling pathway. More interestingly, further analysis found that ADAM12 expression was positively correlated with the immune infiltration levels of B cells, CD8^+^T cells, and CD4^+^T cells, and similarly, TIMER2.0 analysis showed that ADAM12 expression was positively correlated with the infiltration levels of CD4^+^T cells, CD8^+^T cells, B cells, neutrophils, macrophages, and dendritic cells. These results suggest that the level of ADAM12 expression may predict the immune infiltration of tumor cells, which may provide some reference value for immunotherapy for ccRCC. ADAM12 expression was also positively correlated with PD-1, PD-L1, and CTLA4 expression in ccRCC. Previous studies showed that patients with a high expression of PD-L1 would be well treated using an anti-PD-L1 approach [60]. These results suggest that ADAM12 may play an essential role in developing renal clear cell carcinoma and immunoregulatory processes and may also affect immune cell infiltration and the outcome of immunotherapy. Therefore, targeting ADAM12 may become an alternative strategy for tumor therapy.

Although this study improved our understanding of the role of ADAM12 in ccRCC, there were several limitations. First, *in vitro* cell assays should be used to inhibit or increase ADAM12 expression to verify whether it plays a regulatory role in ccRCC, and second, we could not identify a direct mechanism by which ADAM12 participated in the development and progression of ccRCC. Thus, further experimental studies are needed to verify our results.

## Conclusion

In conclusion, our results demonstrated that ADAM12 expression was elevated in ccRCC and that the high expression of ADAM12 was an independent poor prognostic factor in ccRCC. ADAM12 could interact with has-miR-204-5P and SNHG4, LINC01232, and FAM30A, constituting a ceRNA network and playing a role in ccRCC development. Furthermore, ADAM12 mediated immune cell infiltration in the tumor microenvironment. This study demonstrated ADAM12 can serve as a prognostic biomarker for ccRCC, highlighting its potential as a predictive biomarker and immunotherapeutic target.

## Highlights

- ADAM12 is a potential oncogene in clear cell renal cell carcinoma
- High expression of ADAM12 might be a risk factor for patients with clear cell renal cell carcinoma
- ADAM12 is a potential diagnostic and prognostic biomarker for clear cell renal cell carcinoma
